# Occupational and educational inequalities in exit from employment at older ages: evidence from seven prospective cohorts

**DOI:** 10.1136/oemed-2017-104619

**Published:** 2018-03-12

**Authors:** Ewan Carr, Maria Fleischmann, Marcel Goldberg, Diana Kuh, Emily T Murray, Mai Stafford, Stephen Stansfeld, Jussi Vahtera, Baowen Xue, Paola Zaninotto, Marie Zins, Jenny Head

**Affiliations:** 1 Department of Epidemiology and Public Health, University College London, London, UK; 2 Department of Biostatistics and Health Informatics, Institute of Psychiatry, Psychology & Neuroscience, King’s College London, London, SE5 8AF, UK; 3 Department of Epidemiology and Public Health, University College London, London, UK; 4 INSERM, Population-based Epidemiologic Cohorts Unit-UMS 011, Villejuif, France; 5 Paris Descartes University, Paris, France; 6 Inserm, Population-based Epidemiologic Cohorts Unit-UMS 011, Villejuif, France; 7 MRC Unit for Lifelong Health and Ageing, University College London, London, UK; 8 Wolfson Institute of Preventive Medicine, Queen Mary University of London, London, UK; 9 Department of Public Health, University of Turku and Turku University Hospital, Turku, Finland; 10 INSERM UMR 1168, VIMA, Villejuif, France

**Keywords:** extended working life, socioeconomic position, health-related work exit

## Abstract

**Objectives:**

Past studies have identified socioeconomic inequalities in the timing and route of labour market exit at older ages. However, few studies have compared these trends cross-nationally and existing evidence focuses on specific institutional outcomes (such as disability pension and sickness absence) in Nordic countries. We examined differences by education level and occupational grade in the risks of work exit and health-related work exit.

**Methods:**

Prospective longitudinal data were drawn from seven studies (n=99 164). Participants were in paid work at least once around age 50. Labour market exit was derived based on reductions in working hours, changes in self-reported employment status or from administrative records. Health-related exit was ascertained by receipt of health-related benefit or pension or from the reported reason for stopping work. Cox regression models were estimated for each study, adjusted for baseline self-rated health and birth cohort.

**Results:**

There were 50 003 work exits during follow-up, of which an average of 14% (range 2–32%) were health related. Low level education and low occupational grade were associated with increased risks of health-related exit in most studies. Low level education and occupational grade were also associated with an increased risk of any exit from work, although with less consistency across studies.

**Conclusions:**

Workers with low socioeconomic position have an increased risk of health-related exit from employment. Policies that extend working life may disadvantage such workers disproportionally, especially where institutional support for those exiting due to poor health is minimal.

**Key messages**

**What is already known about this subject?**

- Low socioeconomic position (SEP) has previously been associated with increased risk of health-related exit from work. However, most existing evidence is based only on Nordic countries and has focused on specific institutional outcomes, such as disability pension and sickness absence.

**What are the new findings?**

- We examine socioeconomic inequalities in the risk of work exit and health-related work exit based on cross-national data from seven studies.
- Workers with low level education or low occupational grade were more likely to leave work for health reasons, compared with workers with high level education or occupational grade, after adjustment for self-rated health and birth cohort.

**How might this impact on policy or clinical practice in the foreseeable future?**

- Policies that extend working life may disadvantage workers with low SEP disproportionally.

## Introduction

Population ageing has made extended working life a policy priority across Europe. To keep welfare states sustainable, many governments are seeking to raise the age of state pension eligibility. However, remaining in work until or beyond pensionable age may be challenging to those of lower socioeconomic position (SEP). Low SEP has been associated with higher levels of morbidity^1^ and lower disability-free life expectancy.^2^ Socioeconomic inequalities over the life course have also been linked to cognitive and physical functioning at older ages,^3^ with individuals from socioeconomically disadvantaged families being at higher risk of experiencing reduced functioning. Since poor health^4^ and reduced physical or cognitive capabilities^5^ are predictive of work exit, socioeconomic inequalities in health may result in unequal opportunities to extend working life. Furthermore, low SEP may amplify the adverse influence of poor health on health-related work exit.^6^

SEP has also been directly linked to labour market outcomes in later life. Individuals with low SEP are at greater risk of leaving work involuntarily, either through disability retirement^7 8^ or unemployment.^9^ SEP may also influence the timing of labour market exit, but past findings have been mixed. Existing studies have shown individuals at both ends of the occupational ladder to be more likely to extend working life,^10^ but for different reasons. Lower SEP individuals may have lower pension contributions and reduced access to private or occupational pension schemes and, therefore, remain in work out of financial necessity. Higher SEP individuals may retire later because they have better health, stronger attachment to work or are sheltered from labour market constraints associated with involuntary exit (such as unemployment). They tend to have more years of education and later labour market entry, compared with those with low SEP, which also leads to later retirement.^11^

Despite good evidence linking low SEP to poor health and involuntary work exit, existing analyses have several limitations. First, with the exception of the Survey of Health, Ageing and Retirement in Europe (SHARE),^12^ most studies have focused on a single country. Second, existing comparative studies have centred on Nordic countries and are not easily generalisable to other European regions. Third, most existing studies have relied on institutional definitions of health-related work exit (such as disability pension and sickness absence) and few have constructed measures of health-related exit that allow comparison across national contexts. Fourth, few studies have assessed multiple subdomains of SEP,^7^ such as both education and occupational grade. The objectives of our study were thus to (a) derive a consistent measure of the age of labour market exit; (b) construct measures of health-related exit appropriate to the national context and policy on health-related early retirement and (c) to test associations with low education and low occupational grade.

We stratify our analyses by sex, following past research highlighting the gendered nature of retirement timing and the influence of prior employment trajectories.^13 14^ Whereas men’s trajectories traditionally involve continuous paid employment, women’s careers are more likely to be interrupted by caregiving or family responsibilities and their retirement decisions are constrained by gendered social and institutional norms.^15^ Career disruptions earlier in life due to family formation or caregiving may lead to reduced earnings and lower pension contributions over the life course,^16^ which may influence retirement decisions. Many pension systems have historically offered earlier retirement ages for women, for example, in the UK, where, until 2010, women were eligible for statutory pension at age 60, compared with 65 for men.

## Methods

### Data

Data were drawn from seven independent studies in Finland, France, the UK and the USA. Each of these studies was part of the Research in Extending Working Lives (renEWL) consortium. Finnish data came from three waves of the Finnish Public Sector study (FPS),^17^ and French data were taken from the GAZEL cohort study.^18^ Data for the UK came from the British Household Panel Survey (BHPS),^19^ the Whitehall II cohort study,^20^ the English Longitudinal Study of Ageing (ELSA)^21^ and the MRC National Survey of Health and Development (NSHD).^22^ Data for the USA were drawn from the Health and Retirement Study (HRS).^23^

The chosen studies differed in their design and population characteristics. The BHPS, ELSA, and HRS are representative of the general population (in ELSA for ages 50+ only) with fieldwork taking place between 1991/1992 and 2014/2015. Whitehall II and the FPS are samples of public sector workers in England and Finland, respectively, with fieldwork between 1991–1994 and 2012–2013. These workers tend to enjoy greater job security and more generous pension entitlements, compared with private sector workers. GAZEL is a sample of employees from Électricité de France-Gaz de France, the French utility company, with baseline in 1989 and follow-up until 2014. These workers enjoy similar benefits to public sector workers. Employees tend to remain within the company throughout their career, receive regular health assessments and are paid disability and retirement benefits by the company itself. Lastly, the NSHD is a nationally representative birth cohort with fieldwork taking place every 2–3 years since 1946. For this study, we used information from 1989 until 2014. For all studies, therefore, we draw on information collected between 1989 and 2015 (see online supplementary table 1).

10.1136/oemed-2017-104619.supp1 Supplementary data

In all studies, the target sample was participants who were in paid employment at or around age 50. The exact criteria for inclusion in each study are detailed in online supplementary table 1. In the general population surveys (BHPS, ELSA and HRS), we selected participants in paid work at least once between ages 45 and 55. In the occupational cohorts (Whitehall II, FPS and GAZEL), we selected employees aged 40–75. For all studies, participants provided informed consent to take part in the original study.

### Age of labour market exit

Age of labour market exit was defined as the participant’s age at their last exit from paid employment, without subsequent re-employment during follow-up. This was based on self-reported retirement age, administrative or company records or derived as the midpoint between the last interview in paid work and the subsequent interview when the participant was no longer working (see online supplementary table 2 for details). Individuals for whom an exit from work was not observed (either because they were still working at the end of follow-up or because they died or left the study before exiting work) were treated as right censored.

### Health-related labour market exit

We considered an exit to be ‘health related’ if one of two conditions was met: (1) the individual reported leaving work (or retiring) due to their own ill health or disability or (2) the individual was receiving a health-related benefit or pension in the 12 months before, or 12 months after, the date they stopped working. The measurement of these criteria varied across the seven studies (see online supplementary table 2).

### Socioeconomic position

SEP was measured by education and occupational grade, both collected at baseline via self-completion questionnaires (in NSHD and ELSA, face-to-face interviews were used instead). Baseline here refers to the first time a participant entered the analytical sample, which may be later than the start of the study (eg, NSHD). Education was measured as highest formal qualification and coded in three categories: low, middle and high. ‘Low’ corresponded to having no education or low level qualifications; ‘middle’ corresponded in most studies to secondary education (eg, qualifications gained at ages 16–18) and ‘high’ corresponded to tertiary level qualifications. Occupational grade was also coded in three categories with ‘low’ representing routine or manual occupations, ‘middle’ representing intermediate or skilled occupations and ‘high’ representing professional or managerial occupations (see online supplementary table 2 for details).

### Covariates

Self-rated health (SRH) was measured at baseline in all studies and recoded as a dummy variable indicating ‘poor health’. In all studies except GAZEL, participants were asked to assess their general health on a five-point scale. ‘Poor health’ was indicated by responses of ‘Poor’ (in ELSA, HRS, NSHD and Whitehall II), ‘Poor’ or ‘Very poor’ (in BHPS) and ‘Fairly Poor’ or ‘Poor’ (in FPS). Participants in GAZEL rated their general health on an eight-point scale (from ‘Very good’ to ‘Very poor’) and the last four categories were grouped as ‘poor health’. The wordings of questions and responses for each cohort are given in online supplementary material. Birth cohort was measured with three categories (early, middle and late) based on tertiles of birth year in each cohort.

### Statistical analysis

We analysed individual-level data for each study using Cox proportional hazards regression models (with age as the timescale), having established that the proportional hazards assumption was not violated. Participants were followed from their age at baseline (when they first became eligible for inclusion in our sample) until their last exit from paid work or until right censoring (ie, the age the participant left the study, died or reached the end of follow-up). Models were estimated for ‘any exit route’ and ‘health-related exit’ in turn. The former included all exit events, whereas the latter focused on health-related exits only, treating all other exit types as censored. All models were adjusted for poor SRH at baseline, following past studies showing poor health to be associated with early or health-related exit from work.^4^ Poor health is more common among individuals with low education or occupational grade,^24^ compared with high, and the association of poor health with health-related exit from work is strongest in low grade occupations.^6^ Retirement outcomes may also be influenced by time-varying sociocultural or institutional factors within country, such as changes to statutory pension ages or attitudes towards retirement. We therefore additionally adjusted for birth cohort. Our analyses were stratified by sex, as described above. All models were estimated in *Stata 14*.^25^

### Sensitivity analyses

We conducted five sensitivity analyses. First, to assess whether our results were influenced by varying study designs, we repeated all models with studies grouped into three categories: General population survey (BHPS, ELSA and HRS), occupational cohort (FPS, GAZEL and Whitehall II), and birth cohort (NSHD). Group differences were tested using interaction terms (eg, ‘low level education’ × group) and statistical significance assessed using Wald tests (χ^2^). Second, using the same approach, we also compared studies according to whether health-related exit was based on administrative data (GAZEL and FPS) or self-report (all other studies). Third, for the BHPS and ELSA, it was possible to compare ascertainment of health exit (ie, based on receipt of health-related benefit or reported reason for stopping). For these studies, we compared results from separate Cox regression models for each measure of health exit. Fourth, recognising that institutional support (eg, pensions or disability benefits) may be more widely available after statutory retirement age, we repeated our analyses with the samples restricted to age ≤65, the median statutory retirement age across European countries during follow-up. Finally, since low occupational grade may be related to poor health, and subsequent work exit through the mediating pathway of poorer health, adjusting for poor health may represent overadjustment. We therefore present all models (i) unadjusted, (ii) adjusted for birth cohort only and (iii) adjusted for birth cohort and poor SRH.

## Results

We excluded from our analyses 3889 (3.8%) of 103 053 participants who had missing data for SRH (n=2239), education (n=1455) or occupational grade (n=477). A total of 99 164 participants were included in the analysis, of whom 61.0% were women. For this analytical sample, the mean age at baseline was 48.0 years (SD 5.5) and the mean follow-up ranged from 6.9 years in FPS to 18.0 years in NSHD (table 1). During 834 716 person-years at risk, there were 50 003 work exits, of which 7012 (14.0%) were health related (this varied between studies, from 2.4% in GAZEL to 31.9% in FPS).

**Table 1:** Descriptive statistics for the analytical sample

Study	Sex	Participants (n)	Mean (range) age at baseline	Mean (range) of years of follow-up	Participants (n) (%) with poor SRH	Participants (n) (%) with low education	Participants (n) (%) with low occupation	Work exits (n) during follow-up	Health-related exits (n) (% of all exits)
BHPS	M	1261	47.1 (45.0–55.0)	7.3 (0.5–17.5)	100 (7.9)	226 (17.9)	552 (43.8)	548	123 (22.4)
	F	1454	46.8 (45.0–55.0)	7.3 (0.6–17.5)	118 (8.1)	332 (22.8)	424 (29.2)	682	180 (26.4)
ELSA	M	1557	52.1 (45.0–55.0)	7.2 (1.0–14.4)	51 (3.3)	344 (22.1)	630 (40.5)	734	94 (12.8)
	F	2300	51.3 (45.0–55.0)	7.0 (0.9–14.5)	68 (3.0)	609 (26.5)	878 (38.2)	1055	146 (13.8)
NSHD	M	1055	43.0 (43.0–43.0)	18.8 (1.0–25.0)	31 (2.9)	413 (39.1)	110 (10.4)	782	114 (14.6)
	F	1084	43.0 (43.0–43.0)	17.2 (1.0–25.0)	18 (1.7)	432 (39.9)	140 (12.9)	949	144 (15.2)
Whitehall II	M	5017	49.1 (39.6–62.9)	11.7 (0.0–22.3)	457 (9.1)	1878 (37.4)	333 (6.6)	3658	292 (8.0)
	F	2151	50.1 (39.6–62.1)	9.8 (0.0–21.9)	297 (13.8)	1244 (57.8)	814 (37.8)	1690	170 (10.1)
FPS	M	11 415	48.8 (40.0–65.0)	6.7 (0.1–11.0)	820 (7.2)	1660 (14.5)	3986 (34.9)	3258	912 (28.0)
	F	45 335	48.2 (40.0–65.0)	6.9 (0.1–11.0)	2068 (4.6)	4824 (10.6)	6731 (14.8)	12 563	4141 (33.0)
GAZEL	M	14 657	45.0 (41.0–50.0)	10.0 (0.0–25.0)	1111 (7.6)	2908 (19.8)	1741 (11.9)	14 657	252 (1.7)
	F	3719	44.3 (40.0–50.0)	11.1 (0.1–25.0)	373 (10.0)	1169 (31.4)	714 (19.2)	3719	177 (4.8)
HRS	M	3724	52.9 (45.1–56.0)	11.2 (2.0–22.7)	315 (8.5)	577 (15.5)	1166 (31.3)	2798	129 (4.6)
	F	4435	51.7 (45.0–56.0)	11.3 (2.0–22.6)	299 (6.7)	610 (13.8)	1018 (23.0)	3293	138 (4.2)

BHPS, British Household Panel; ELSA, English Longitudinal Study of Ageing; FPS, Finnish Public Sector Study; GAZEL, Electricité De France-Gaz De France; HRS, Health and Retirement Study; NSHD, National Survey of Health and Development; SRH, self-rated health.

A total of 17 226 (17.4%) participants had low level education at baseline. This varied considerably across studies, partly due to differences in birth cohort, from 11.4% in FPS to 43.6% in Whitehall II. There were 19 237 (19.4%) participants in low grade occupations and 6126 (6.2%) with poor SRH at baseline. Across all studies, poor health was more common among individuals with low compared with high occupational grade (9.2% vs 4.7%) and with low level education (9.1% vs 4.7%), consistent with past research.^24^ Women were on average less likely than men to have low level education (15.2% vs 20.7%) or low occupational grade (17.7% vs 22%), but this varied by study. Sex differences in low education were small (<10%) in all studies except Whitehall II, where women were considerably more likely than men to have low level education (57.8 and 37.4 per cent, respectively). Sex differences in occupational grade were more substantial. In Whitehall II, women were much more likely to be in low grade employment than men (37.8% vs 6.6%), and male employees in the FPS were more likely than female employees to be in low grade occupations (34.9% vs 14.8%). These differences may reflect the nature of civil service or public sector recruitment, compared with the private sector.

### Any exit

**Figure 1:**
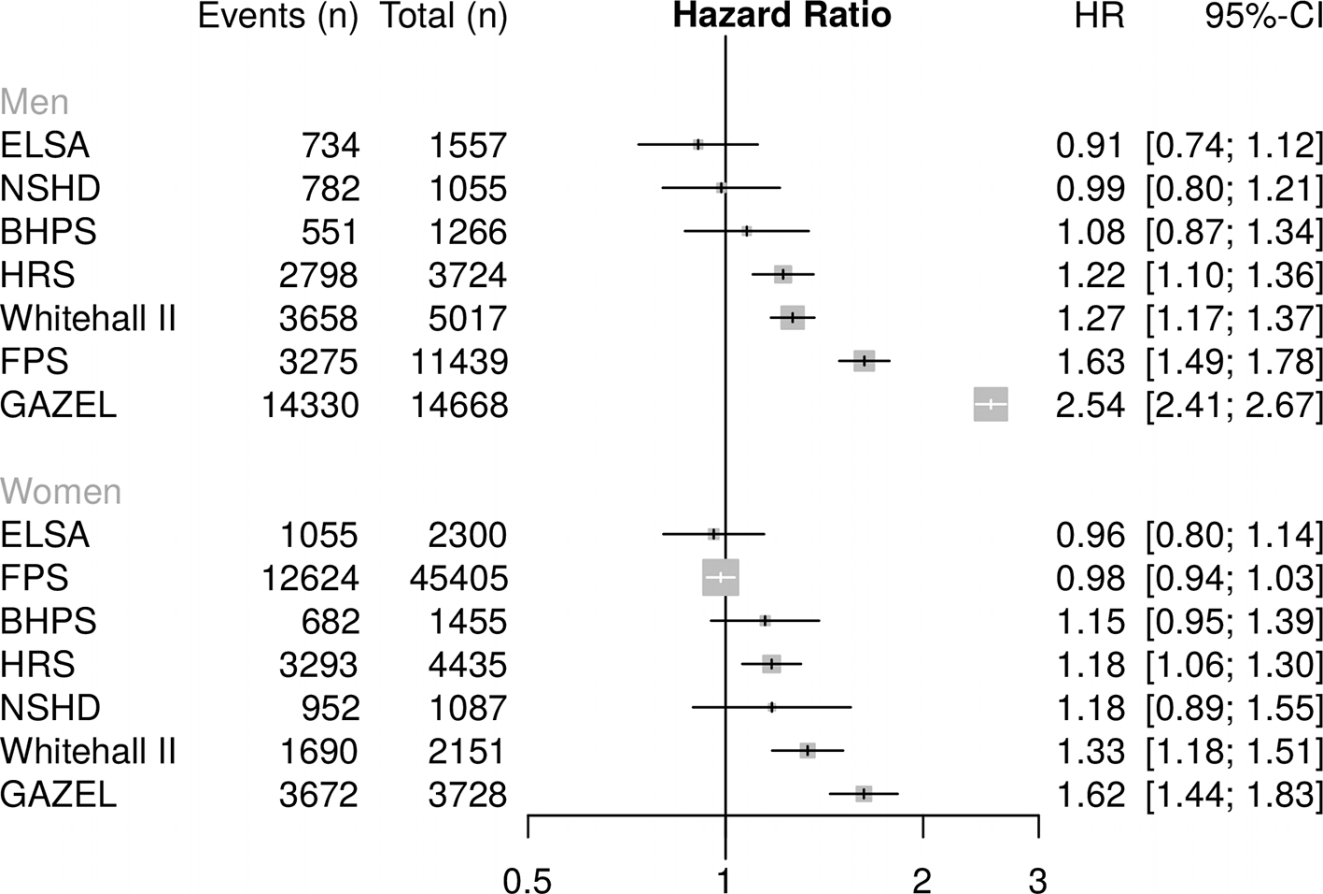
Association of low level education with risk of any work exit. BHPS, British Household Panel; ELSA, English Longitudinal Study of Ageing; FPS, Finnish Public Sector Study; GAZEL, Electricit é De France-Gaz De France; HRS, Health and Retirement Study; NSHD, National  Survey of Health and Development.

**Figure 2:**
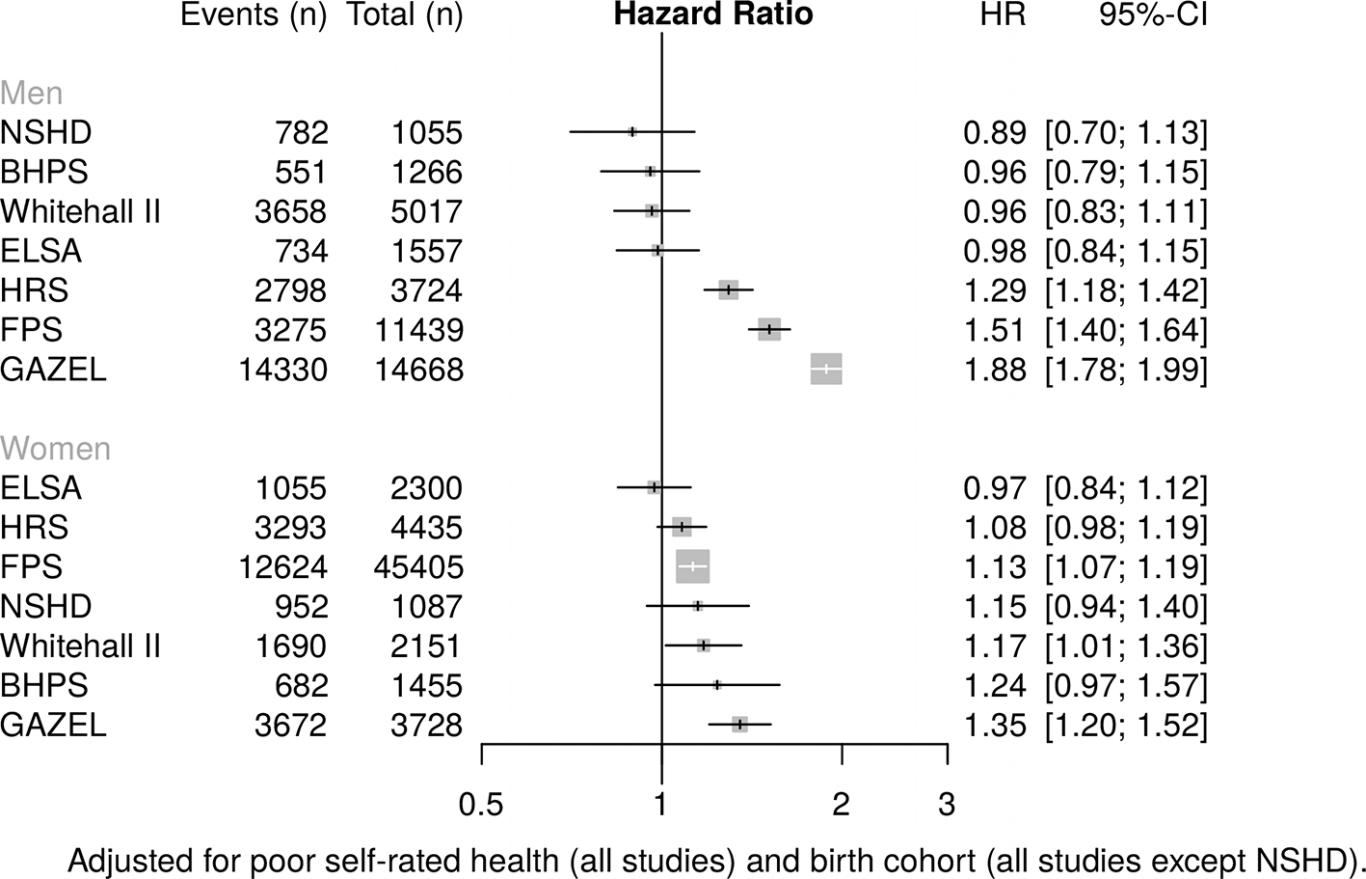
Association of low occupational grade with risk of any work exit. BHPS, British Household Panel; ELSA, English Longitudinal Study of Ageing; FPS, Finnish Public Sector Study; GAZEL, Electricit é De France-Gaz De France; HRS, Health and Retirement Study; NSHD, National Survey of Health and Development.

Figures 1 and 2 present associations of low education and low occupational grade with risk of exit from paid work (for all routes, including health-related exit), stratified by sex and adjusted for poor SRH and birth cohort. There was considerable heterogeneity in the findings. Low level education (figure 1) was associated with an increased risk of exit from paid work in 5/7 studies for men and women, though this did not attain statistical significance in some. For women, significantly raised HRs were observed in the HRS, Whitehall II and GAZEL, ranging from 1.18 (95% CI 1.06 to 1.30) in HRS to 1.62 (95% CI 1.44 to 1.83) in GAZEL; for men, in the HRS, Whitehall II, FPS and GAZEL, with HRs ranging from 1.22 (95% CI 1.10 to 1.36) in HRS to 2.54 (95% CI 2.41 to 2.67) in GAZEL. Low occupational grade (figure 2) was associated with significantly increased risk of work exit in three studies for both women (FPS, Whitehall II and GAZEL) and men (HRS, FPS and GAZEL). HRs for women ranged from 1.13 (95% CI 1.07 to 1.19) in FPS to 1.35 (95% CI 1.20 to 1.52) in GAZEL; for men from 1.29 (95% CI 1.18 to 1.42) in HRS to 1.88 (95% CI 1.78 to 1.99) in GAZEL. In some studies, middle level education (compared with high) was associated with increased risk of exit from work (see online supplementary table 3), but these associations were weaker and some not statistically significant.

### Health-related exit

**Figure 3:**
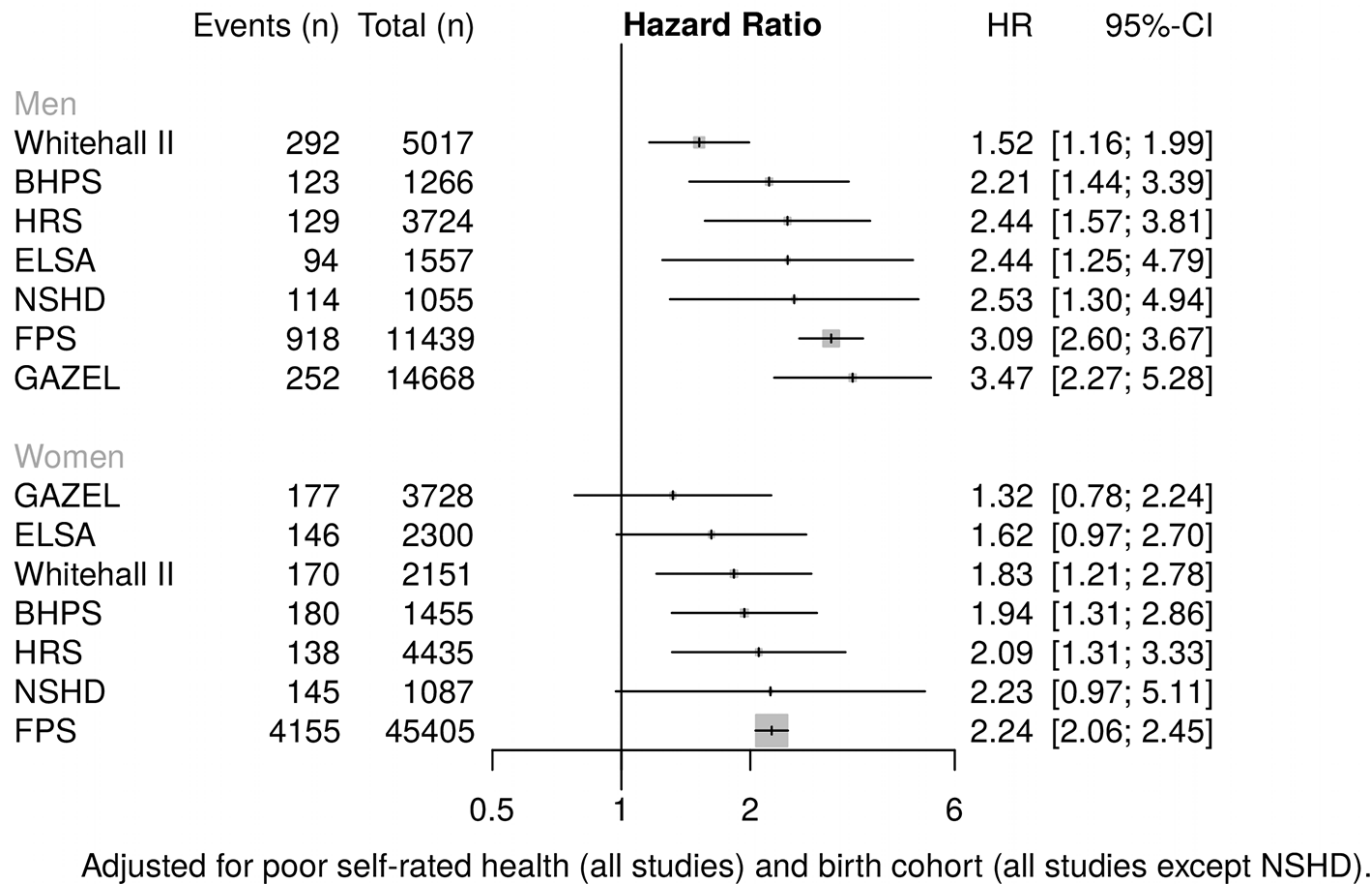
Association of low level education with risk of health-related work exit. BHPS, British Household Panel; ELSA, English Longitudinal Study of Ageing; FPS, Finnish Public Sector Study; GAZEL, Electricit é De France-Gaz De France; HRS, Health and Retirement Study; NSHD, National Survey of Health and Development.

**Figure 4:**
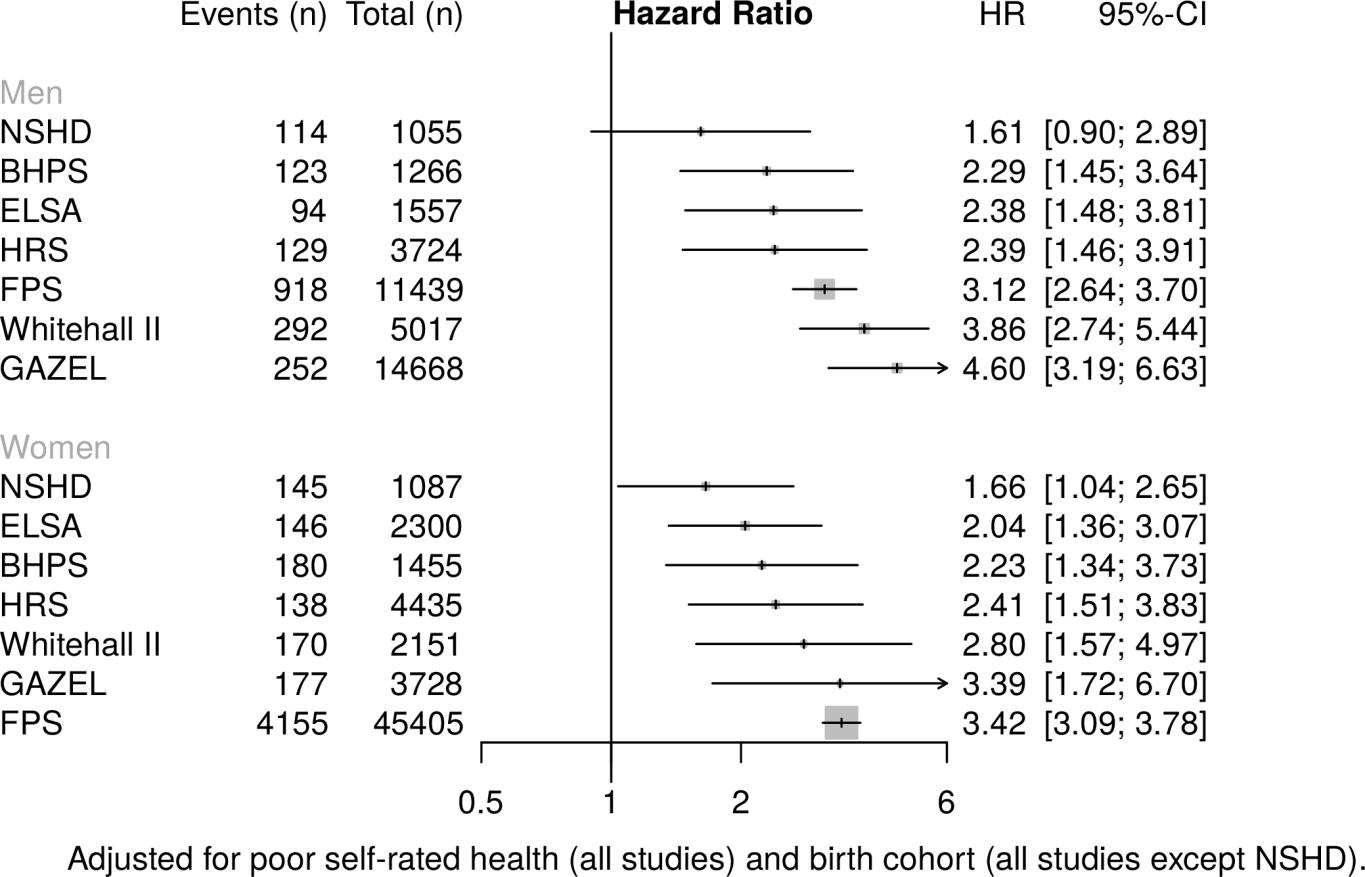
Association of low occupational grade with risk of health-related work exit. BHPS, British Household Panel; ELSA, English Longitudinal Study of Ageing; FPS, Finnish Public Sector Study; GAZEL, Electricit é De France-Gaz De France; HRS, Health and Retirement Study; NSHD, National Survey of Health and Development.

The results for health-related exit from work (figures 3 and 4) were more consistent. A positive, statistically significant association between low level education and health-related exit from work was observed for men in all studies, and for women in all studies except ELSA and NSHD. For women in ELSA and NSHD, the association was in the same direction but borderline significant. Statistically significantly raised HRs for women ranged from 1.83 (95% CI 1.21 to 2.78) in Whitehall II to 2.24 (95% CI 2.06 to 2.45) in FPS. For men, from 1.52 (95% CI 1.16 to 1.99) in Whitehall II to 3.47 (95% CI 2.27 to 5.28) in GAZEL.

Low occupational grade was strongly associated with health-related exit from work in all studies, although it did not attain statistical significance for men in NSHD. For women, HRs ranged from 1.66 (95% CI 1.04 to 2.65) in NSHD to 3.42 (95% CI 3.09 to 3.78) in FPS; for men from 2.29 (95% CI 1.45 to 3.64) in BHPS to 4.60 (95% CI 3.19 to 6.63) in GAZEL. Middle occupational grade (compared with high) was associated with increased risk of health-related exit in 3/7 and 5/7 studies for women and men, respectively (see online supplementary table 6).

### Sensitivity analyses

There were some differences in the risk of work exit based on study design (see online supplementary table 7), but these were largely driven by differences between the birth cohort (NSHD) and other studies. Where statistically significant Wald χ^2^ statistics were observed (indicating a difference by study design), the HR for NSHD tended to not attain statistical significance, whereas the HRs for ‘general population survey’ and ‘occupational cohort’ tended to be similar and have overlapping CIs. There were two exceptions. First, men with low occupational grade in occupational cohorts were at greater risk of health-related exit (HR=4.71; 95% CI 4.12 to 5.39) compared with men with low occupational grade in the general population surveys (2.57; 95% CI 1.96 to 3.38). Second, women with low level education in general population surveys were at greater risk of health-related exit (2.75; 95% CI 2.14 to 3.52) compared with women in occupational cohorts (1.79; 95% CI 1.65 to 1.94).

We observed stronger associations between low occupational grade and health-related exit when health exit was assessed based on register data rather than self-report, but in all cases the associations were positive and statistically significant (see online supplementary table 7).

Changing the measurement of ‘health-related exit’ in the BHPS did not influence our results. Associations of low education and low occupational grade with the risk of health exit were similar whether based on the ‘reason for stopping work’ or the ‘receipt of health-related benefit or pension’. However, in ELSA, the association of low occupational grade with the risk of health-related work exit was higher when health exit was based on the ‘reason for stopping work’ measure (HR=3.41 (95% CI 1.76 to 6.62) and 11.56 (95% CI 3.54 to 37.79) for women and men, respectively), compared with when using the measure of ‘receipt of health-related benefit’ (HR=1.56 (95% CI 1.01 to 2.41) and 2.23 (95% CI 1.33 to 3.76) for women and men, respectively).

When we restricted the analytical sample to ages 40–65, most estimates were unchanged (see online supplementary tables 3–6), but there were several important differences. Some associations were attenuated to non-significance when estimated for the restricted sample (eg, the association between low level education and risk of health-related exit for men in Whitehall II). In other cases, we found the reverse, that is, positive associations only attained statistical significance when estimated for the restricted sample. Notably, among Whitehall II participants aged 40–75, whereas the association between low occupational grade and ‘any exit’ from work was positive or not statistically significant (for women and men, respectively), when estimated for ages 40–65 these associations were negative and statistically significant. For Whitehall II participants at or below statutory retirement age, therefore, low occupational grade (compared with high) was associated with reduced risk of exit from work.

Finally, there were no substantive differences when comparing results that were unadjusted, adjusted for birth cohort only or adjusted for birth cohort and poor SRH (see online supplementary tables 3–6).

## Discussion

For the first time, prospective individual participant data from seven studies (n=99 164) were used to test associations of low education and low occupational grade with the risks of work exit in older age. We found strong socioeconomic inequalities in the risk of health-related work exit. After adjustment for poor SRH and birth cohort, low education and low occupational grade were associated with an increased risk of leaving work for health reasons in 6/7 studies. Older workers with low SEP were also more likely to stop working for any reason (whether health-related or not), but the results were more heterogeneous and did not attain statistical significance in all studies.

This analysis builds upon past evidence highlighting inequalities in labour market outcomes in later life. Our study is one of the first to assess health-based exit by combining individual accounts (ie, the ‘reason for stopping work’) with information on receipt of health-related social transfers. It is also one of few studies to examine health-related exit using longitudinal data from outside Nordic countries, to compare multiple countries or to assess both ‘any exit’ and ‘health-related’ exits from work.

Our main findings are consistent with previous studies of socioeconomic and educational differences in labour market exit. A register-based study in Finland found a higher risk of disability retirement among men and women with low education and low SEP.^6 7^ Educational differences in work exit were found among Dutch workers^9^ and educational differences in disability pension were found in a study of Swedish men.^8^ This has important implications for current policies regarding extended working life. Many Western countries are seeking to increase state pension ages, in line with rising life expectancy. Some countries, including the UK, have introduced legislation to remove compulsory retirement ages. Retirement age is typically calculated by sex and rarely takes into account other factors such as family circumstances or SEP. Our results suggest that the opportunities for extended working may depend on SEP, whereby older workers are more likely to leave work due to poor health if they are less educated or work in manual or routine occupations, compared with those with tertiary qualifications or professional occupational groups. Policies which extend working life, therefore, are likely to disadvantage such workers disproportionally—especially in countries where institutional support for health-based work withdrawal is minimal.

Less consistent findings regarding the risk of ‘any exit’ from work may reflect contrasting ‘push’ and ‘pull’ influences on the decision to continue working. On the one hand, workers with low SEP are more likely to experience poor health or disability, compared with those with high SEP, and thus be forced to exit the labour market early. On the other hand, these workers are more likely (compared with those with higher SEP) to lack the necessary pension contributions or financial resources needed to retire^26^ and may be forced to keep working. Conversely, highly educated workers in upper occupational groups may have the financial resources that would enable early retirement, but may opt to continue working due to high levels of organisational commitment^27^ or job satisfaction.^28^ They may also be better placed to continue working even with a health condition, for example, by making necessary changes to their work environment or arrangements.

In some studies, we found no association between low SEP and work exit, which may be attributable to sample selection. We restricted our analysis to participants who were working at least once at ages 45–55 and omitted those who left the labour market earlier or never worked at all. Lifetime SEP has previously been linked to health^29^ and employment^30^ outcomes in later life. Our sample is likely to be healthier and better educated than participants who exited the labour market before age 45. Low SEP is associated with poor health and non-employment in midlife, which in turn predict early labour market exit. Low SEP may influence retirement timing via outcomes in midlife and by selecting a sample of older workers we may be underestimating the influence of SEP on retirement timing.

Alternatively, heterogeneous results may reflect contextual differences between the UK and other countries, for example, with regard to statutory pension age or eligibility. For men outside the UK, low SEP was consistently associated with an increased risk of ‘any exit’, whereas no association was found in most British studies. The included studies varied markedly in their design, ascertainment of age of exit from paid work and route of exit, measurement of covariates and population characteristics. Some were nationally representative, whereas other captured particular occupational sectors (the public sector in Finland, the GAZEL study of gas and electricity employees and civil servants in the Whitehall II study). Such heterogeneity requires that our findings are interpreted on a study-by-study basis. Low education and low occupational position were predictive of health-related exit in most studies, but with varying strengths of association. These differences must be interpreted within the institutional and policy context of each study. Factors such as statutory pension age, delayed retirement incentives or the availability and generosity of disability retirement schemes will shape routes out of the labour market.

### Limitations

Strengths of our study included the long follow-up, adjustment for a harmonised measure of SRH, the inclusion of a range of study types across several countries and the combination of self-report and administrative data to ascertain health exit. Past studies have shown low education or low occupational grade to predict disability pension^7^ or sickness absence,^31^ but most have relied on administrative definitions of ill health.

Deriving a consistent measure of health-related exit is a major challenge for cross-national research, given national differences in retirement and social security policies. However, despite such policy differences, it is of interest to compare socioeconomic inequalities in health-related exit among different cohorts. While we identified a measure of health-related exit for all countries, we cannot rule out the possibility that observed differences in health-related exit may partly reflect the use of inconsistent measures.

The measurement of ‘health-related’ work exit was based on receipt of health-related social transfer and/or the reported reason for exit. Policies regarding disability pension or benefit vary considerably across countries in terms of generosity, eligibility and coverage. An exit from work was more likely to be identified as health-based if the individual lived in a country with lower eligibility criteria. Our analysis ascertained receipt of health benefit based on either self-report or linkage to administrative records. Individuals may under-report benefit receipt^32^ or fail to take up the available support, and these individuals may differ systematically from those who do receive benefits.^33^ Policy change within countries, over time, may also influence uptake of benefits.^34^

Our analysis focused on exit from paid work rather than retirement, to avoid reproducing institutional definitions regarding statutory retirement that vary across countries and over time. However, ascertaining final labour market exit is difficult. Except in the case of death, individuals can always return to work, following an initial exit, although re-employment becomes less likely with age.^35^ Where studies had linkage to national or occupational employment registers (eg, FPS), it was possible to follow respondents beyond the fieldwork period, thus final labour market exit could be reliably determined. By contrast, in other cohorts, where participants may leave the study after an initial work exit, we cannot be certain that individuals do not later return to work. Past studies suggest unretirement (ie, re-employment after an initial exit) rates of around 20 per cent.^36^ However, since some participants left work more than once, the proportion re-entering the labour market after the end of follow-up is likely to be lower.

To mitigate these problems, we combined benefit receipt with information on the ‘reason for stopping work’. However, reporting of health and disability has been shown to vary considerably between countries^37^ and by education,^38^ with more highly educated individuals tending to assess health more negatively. Health reporting may also be influenced by economic incentives, particularly among disabled or retired respondents, or where social transfers are conditional on poor health. At the same time, however, our findings regarding health-related exit were remarkably consistent between the studies and sensitivity analyses showed similar results when exit route was ascertained by register linkage or self-report.

We were unable to examine inequalities in other routes of exit, such as unemployment or ‘homemaker’, due to small numbers and because these exit types were not measured in all studies. Although we adjusted for SRH, we were unable to adjust for objective health status at baseline and in several studies we relied solely on self-reported measures of work status. Future studies should consider incorporating electronic medical records or administrative data instead. Related to this, we did not adjust for other potential confounders, such as marital status or partner’s employment status. Partly, this was due to difficulties achieving harmonised measures, but more importantly, the aim of our study was to describe socioeconomic inequalities in work exit, not to explain them.

A further limitation is that our analyses did not take account of institutional differences, such as pension availability or retirement policies which are complex and vary between countries^39^ and by occupational sector within countries. As well as influencing the average age of exit, these pension differences may be related to socioeconomic inequalities in age of exit. Investigating the role of pension and retirement policy would require inclusion of studies from additional countries as well as data on eligibility and extent of company pensions or quasi-experimental studies that evaluate the influence of changes in pension policy on labour market participation.

To conclude, we found inequalities in the risk of leaving work for health reasons according to levels of education and occupational grade. These differences were larger in some studies than others and generally were higher in the occupational cohorts (FPS, GAZEL and Whitehall II) compared with the general population surveys, which may reflect better access to sickness absence or disability pension among public sector workers. Our results for ‘any exit’ were less consistent, perhaps reflecting that this outcome incorporates various reasons for stopping work—both push (eg, lay-offs) and pull (eg, pension eligibility). By contrast, low education and low occupational grade were associated with an increased risk of health exit for men and women in almost all studies. This calls for greater flexibility in polices that extend working life and recognition of the barriers to continued employment, such as poor health. Socioeconomic inequalities in health may result in different abilities to work to current pensionable age, thus policies that universally extend working life may disadvantage workers in poorer health. Future research should expand to include a wider range of countries and address the limitations listed above, most notably, the reliance on self-reported assessments of health status and labour market exit.
