# Supplementary material for: Occupational and educational inequalities in exit from employment at older ages: evidence from seven prospective cohorts
Source: Occup Environ Med. 2018 Mar 12;75(5):369–77. doi: 10.1136/oemed-2017-104619 (PMC5909745; doi:10.1136/oemed-2017-104619)
Supplement: Supplementary data [file oemed-2017-104619supp001.pdf]

Supplementary Table 1: Study design and inclusion criteria

Study	Country	Original study baseline	Year first included in analytical sample	Sample	Response rate at study baseline (%)	N (total sample)	Data collection	End of follow-up	N (included in analyses)	Criteria for inclusion in the analytical sample
BHPS	United Kingdom	1991-92	1991-92	Individuals living in private households in Great Britain (excluding Northern Ireland and north of the Caledonian canal).	92	10,264	Annually.	2008	2,715	Members of the original BHPS cohort (baseline 1991/2) who were in paid work (>0 hours/week) on at least one occasion at ages 45-55.
Understanding Society	United Kingdom	2010-12	2009-10	BHPS participants who remained in the study until 2008 were invited to join the Understanding Society panel, with interviews first taking place in 2010/12.	64	10,264†	Annually.	2015	2,715	Members of the BHPS sample who had not stopped working or left the study by 2008.
ELSA	United Kingdom	2002-03	2002-03	Individuals aged 50+ living in private households in England.	66	16,085	Biennially.	2014-15	3,857	Participants who were in paid work (>0 hours/week) and aged 45-55 at least once during follow-up, with complete information.
NSHD	England, Scotland, and Wales	1946	1989	Birth cohort based on a nationally representative, social class stratified sample of singleton births that took place in one week in March 1946 in England, Scotland and Wales.	78-95	5,362	At birth (from the mother) and 24 times thereafter, most recently in 1989 (age 43), 1999 (age 53), 2006-10 (age 60-64), and 2014 (age 68).	2014	2,139	Participants who self-report as working (and not already retired) at age 43 or 53, and had complete information.
Whitehall II	England	1991-94	1991-94	British civil service workers from 20 Whitehall departments.	78-87	10,308	Every 2-3 years (most recently in 1995/96, 1997/99, 2001, 2002/04, 2006, 2007/09 and 2012/13).	2012-13	7,168	Participants aged 40-65 and still working at study baseline, with complete information.
FPS	Finland	1997/8	2000	Employees with month job contract in any year from 1991-2005 in 10 towns and five hospital districts in Finland.	82	151,901	2000, 2004, 2008, with register follow-up until 31st December 2011.	2011	56,750	Participants aged 50-75 at the first wave (1997/8), who were not already retired, and had complete information.

GAZEL	France	1989	1997	Cohort Study among Électricité de France-Gaz de France (EDF-GDF; the French national utility company) workers aged 35-50 years.	44	20,625	Annually.	2014	18,376	Employees aged 40-55 at the first wave (1989) who had complete information.
HRS	United States	1992	1992	The initial cohort was a nationally representative sample of individuals aged 51-61 living in private households in the USA. Refreshment samples were added in 1993, 1998, and 2004 and 2010.	81	20,625	Biennially.	2014	8,159	Participants who were in paid work (>0 hours/week) and aged 45-55 at least once during follow-up, with complete information.

BHPS=British Household Panel; ELSA=English Longitudinal Study of Ageing; NSHD=National Survey of Health and Development; FPS=Finnish Public Sector Study; GAZEL=Electricité De France-Gaz De France; HRS=Health and Retirement Study.

Supplementary Table 2: Measurement of outcomes and covariates

	Measurement of outcomes		Measurement of covariates	
Study	*Age of exit*	*Health-related work exit*	*Education*	*Occupational grade*
BHPS and Understanding Society	The participant's age at the midpoint between their last interview in paid work, and their subsequent interview, when they were no longer working. Work status was assessed based on self-reported working hours (0 hours/week = Not working; >0 hours/week = Working).<br><br>If respondents additionally provided the year and month that their last job ended, age of work exit was derived based on this information instead.	Either: receipt of health-related benefit or pension within 12 months of stopping work (Incapacity Benefit, Severe Disablement Allowance, Disability Living Allowance, Attendance Allowance, Industrial Injury Disablement Benefit, Sickness and accident insurance, or other disability-related benefit).<br><br>Or: own poor health reported as the reason for stopping work. Respondents were asked to choose from a card "the statement that best describes why [they] stopped doing that job?" Responses of "Health reasons" were considered to indicate health-related exit.	Based on self-report and categorised as 'low' (no educational qualifications), 'middle' (A/O level or equivalent[†]), and 'high' (tertiary level qualification, such as degree or diploma).	Based on Socio-economic Group (SEG) and catergorised as low (personal service workers, own account workers, agricultural workers, and 'other'), middle (intermediate, junior non-manual workers) or high SEP (managers, professional workers).
ELSA	As for BHPS, above.	As for BHPS, above, except the question wording was "What were your reasons for leaving your previous job?" and responses of "Own ill health or disability" were considered to indicate health-related exit.	As for BHPS, above.	Based on the National Statistics Socio-Economic Classification (NS-SEC) and categorised as low (Routine and manual occupations), middle (Intermediate occupations) or high SEP Managerial and professional occupations).
NSHD	Based on interviews with participants at ages 53, 60-64, and 68-69. Respondents were asked the age at which they "retired from their main occupation" and whether they were currently doing any paid work. For respondents who retired during follow-up, and did not return to work, we took the retirement age as the age of work exit. Respondents who returned to work after retirement from their main occupation were treated as right-censored.	Reason for retiring (reported at interviews at age 53, 60-64 or 68-69) given as "Health reasons".	As for BHPS, above.	Based on Adult Social Class and categorised as low (partly skilled/unskilled occupations), middle (skilled manual, skilled non-manual, intermediate occupations) or high SEP (professional or managerial occupations).

Whitehall II	The participant's age at mid-point between consecutive interviews. Participants were considered to be working if they remained employed in the civil service or were in paid employment elsewhere.	Either: reason for stopping work is reported as "long-term sick"; or if participants had left the civil service, main route of exit was reported as "retirement on health grounds".	Based on self-report and categorised as 'low' ('O-level[†]' or lower), 'middle' ('A-level[†]' or equivalent) or 'high' (Degree level or higher).	Based on a six-level civil service employment grade on the basis of salary. This was categorised as low ('Clerical/Support'), middle ('Profes-sional/Executive') and high SEP ('Administrative').
FPS	Age at receipt of pension (full-time, permanent, or fixed-term), based on national pension records held by the Finnish Centre for Pensions. These nation-wide pension data were available for all participants regardless of their employment status or workplace at the follow-up.	Receipt of disability pension, based on national pension records held by the Finnish Centre for Pensions.	Based on records from Statistics Finland, categorised as 'low' (primary), 'middle' (secondary) or 'high' (tertiary).	Based on occupational titles from employers' registers and categorized according to the classification of occupations by the Statistics Finland [30] into low ('manual'), middle ('lower grade non-manual') and high SEP ('higher grade non-manual').
GAZEL	Age of retirement from company, based on administrative records from the Électricité de France-Gaz de France (EDF-GDF) company.	Receipt of disability pension or long-term sickness absence (600 sick-leave days during two consecutive years).	Based on self-report and categorised as 'low' ('Basic education certificate' or 'Junior secondary certificate'), 'middle' ('Baccalaureate', 'Certificate of professional competence', or 'Vocational certificate') or 'high' (tertiary qualification, such as degree or diploma).	Based on occupational grade from employers' registers and categorised according to a three level classification: low (white and blue-collar), middle (foremen) and high (executive).
HRS	As for BHPS, above.	Receipt of health-related social security payment at the first interview after stopping work. Respondents were asked whether they were receiving Social Security disability insurance (SSDI) or Supplemental Security Income (SSI), the two programs through which disability benefits are administered in the United States.	Based on self-report and categories as 'low' (did not finish high school), 'middle' (completion of high school or GED qualification) and 'high' (tertiary level qualification).	Based on 1980 Census codes and categorised as low ('Manual'), middle ('Lower grade non-manual') or high ('Higher grade non-manual') occupational position.

BHPS=British Household Panel; ELSA=English Longitudinal Study of Ageing; NSHD=National Survey of Health and Development; FPS=Finnish Public Sector Study; GAZEL=Electricité De France-Gaz De France; HRS=Health and Retirement Study.

[†] GCE A Level (Advanced Level) is a subject-based qualification, exams for which are taken by students in the United Kingdom at age 17/18. GCE O Level (Ordinary Level) was a qualification typically obtained at age 16 (it was replaced in 1998 by the General Certificate of Secondary Education (GCSE).

Supplementary Table 3: Hazard ratios (HR) for low education (vs. high) and risk of 'any exit' from work

		**Full sample (ages 40-75)**			**Restricted sample (ages 40-65)**		
		Unadjusted	+ adjustment for birth cohort	+ adjustment for poor self-rated health	Unadjusted	+ adjustment for birth cohort	+ adjustment for poor self-rated health
		HR [95% C.I.]	HR [95% C.I.]	HR [95% C.I.]	HR [95% C.I.]	HR [95% C.I.]	HR [95% C.I.]
BHPS	F	1.14 [0.94, 1.37]	1.20 [0.99, 1.45]	1.15 [0.95, 1.39]	1.17 [0.97, 1.41]	1.22 [1.01, 1.48]	1.18 [0.98, 1.43]
	M	1.06 [0.85, 1.31]	1.10 [0.89, 1.37]	1.08 [0.87, 1.34]	0.91 [0.73, 1.14]	0.94 [0.75, 1.17]	0.91 [0.72, 1.13]
ELSA	F	0.94 [0.79, 1.12]	0.99 [0.83, 1.18]	0.96 [0.80, 1.14]	0.99 [0.82, 1.19]	1.01 [0.84, 1.22]	1.01 [0.84, 1.22]
	M	0.91 [0.74, 1.12]	0.93 [0.76, 1.15]	0.91 [0.74, 1.12]	0.90 [0.72, 1.13]	0.89 [0.71, 1.12]	0.88 [0.70, 1.10]
FPS	F	1.02 [0.97, 1.07]	1.00 [0.96, 1.05]	0.98 [0.94, 1.03]	0.99 [0.94, 1.04]	0.98 [0.93, 1.03]	0.95 [0.91, 1.00]
	M	1.69 [1.55, 1.85]	1.69 [1.54, 1.84]	1.63 [1.49, 1.78]	1.71 [1.57, 1.87]	1.70 [1.56, 1.86]	1.62 [1.48, 1.77]
GAZEL	F	1.61 [1.43, 1.81]	1.62 [1.44, 1.83]	1.62 [1.44, 1.83]	1.64 [1.46, 1.85]	1.66 [1.47, 1.87]	1.66 [1.48, 1.87]
	M	2.52 [2.39, 2.66]	2.54 [2.41, 2.68]	2.54 [2.41, 2.67]	2.55 [2.42, 2.68]	2.57 [2.44, 2.71]	2.57 [2.43, 2.71]
HRS	F	1.09 [0.98, 1.20]	1.23 [1.11, 1.37]	1.18 [1.06, 1.30]	1.33 [1.17, 1.51]	1.23 [1.08, 1.40]	1.21 [1.06, 1.37]
	M	1.11 [1.00, 1.23]	1.27 [1.14, 1.41]	1.22 [1.10, 1.36]	1.15 [1.00, 1.32]	1.03 [0.90, 1.19]	0.99 [0.86, 1.15]
NSHD	F	1.19 [0.90, 1.57]	1.19 [0.90, 1.57]	1.18 [0.89, 1.55]	0.98 [0.73, 1.31]	0.98 [0.73, 1.31]	0.97 [0.72, 1.30]
	M	1.00 [0.82, 1.23]	1.00 [0.82, 1.23]	0.99 [0.80, 1.21]	0.65 [0.52, 0.81]	0.65 [0.52, 0.81]	0.64 [0.51, 0.80]
Whitehall II	F	1.38 [1.23, 1.56]	1.33 [1.18, 1.51]	1.33 [1.18, 1.51]	1.23 [1.08, 1.39]	1.10 [0.96, 1.26]	1.11 [0.97, 1.26]
	M	1.28 [1.18, 1.38]	1.27 [1.17, 1.37]	1.27 [1.17, 1.37]	1.21 [1.12, 1.32]	1.12 [1.03, 1.21]	1.12 [1.03, 1.21]

BHPS=British Household Panel; ELSA=English Longitudinal Study of Ageing; NSHD=National Survey of Health and Development; FPS=Finnish Public Sector Study; GAZEL=Electricité De France-Gaz De France; HRS=Health and Retirement Study.

†Analyses involving NSHD, a birth cohort, were not adjusted for birth cohort.

Supplementary Table 4: Hazard ratios (HR) for low occupational grade (vs. high) and risk of 'any exit' from work

		Full sample (ages 40-75)			Restricted sample (ages 40-65)		
		Unadjusted	+ adjustment for birth cohort	+ adjustment for poor self-rated health	Unadjusted	+ adjustment for birth cohort	+ adjustment for poor self-rated health
		HR [95% C.I.]	HR [95% C.I.]	HR [95% C.I.]	HR [95% C.I.]	HR [95% C.I.]	HR [95% C.I.]
BHPS	F	1.27 [1.00, 1.61]	1.29 [1.01, 1.63]	1.24 [0.97, 1.57]	1.31 [1.03, 1.67]	1.33 [1.04, 1.69]	1.29 [1.01, 1.65]
	M	0.97 [0.80, 1.17]	0.96 [0.79, 1.15]	0.96 [0.79, 1.15]	0.93 [0.76, 1.13]	0.92 [0.76, 1.12]	0.91 [0.75, 1.11]
ELSA	F	0.95 [0.83, 1.09]	0.99 [0.86, 1.13]	0.97 [0.84, 1.12]	1.00 [0.86, 1.16]	1.01 [0.87, 1.17]	0.99 [0.85, 1.15]
	M	0.97 [0.83, 1.13]	1.00 [0.85, 1.17]	0.98 [0.84, 1.15]	0.88 [0.74, 1.05]	0.86 [0.72, 1.02]	0.85 [0.71, 1.01]
FPS	F	1.15 [1.10, 1.21]	1.15 [1.09, 1.21]	1.13 [1.07, 1.19]	1.10 [1.05, 1.16]	1.10 [1.04, 1.15]	1.07 [1.02, 1.13]
	M	1.60 [1.48, 1.73]	1.60 [1.48, 1.73]	1.51 [1.40, 1.64]	1.57 [1.45, 1.70]	1.57 [1.45, 1.70]	1.49 [1.37, 1.61]
GAZEL	F	1.33 [1.18, 1.50]	1.34 [1.19, 1.51]	1.35 [1.20, 1.52]	1.33 [1.18, 1.49]	1.34 [1.19, 1.51]	1.35 [1.20, 1.52]
	M	1.88 [1.78, 1.99]	1.88 [1.78, 1.99]	1.88 [1.78, 1.99]	1.91 [1.80, 2.02]	1.91 [1.80, 2.02]	1.91 [1.80, 2.02]
HRS	F	1.08 [0.98, 1.18]	1.11 [1.01, 1.22]	1.08 [0.98, 1.19]	1.26 [1.12, 1.42]	1.22 [1.08, 1.37]	1.21 [1.07, 1.36]
	M	1.26 [1.15, 1.38]	1.34 [1.22, 1.47]	1.29 [1.18, 1.42]	1.24 [1.10, 1.40]	1.17 [1.04, 1.33]	1.14 [1.01, 1.29]
NSHD	F	1.16 [0.96, 1.41]	1.16 [0.96, 1.41]	1.15 [0.94, 1.40]	0.94 [0.76, 1.15]	0.94 [0.76, 1.15]	0.93 [0.76, 1.14]
	M	0.89 [0.70, 1.14]	0.89 [0.70, 1.14]	0.89 [0.70, 1.13]	0.76 [0.58, 0.99]	0.76 [0.58, 0.99]	0.76 [0.58, 0.99]
Whitehall II	F	1.27 [1.10, 1.47]	1.19 [1.03, 1.38]	1.17 [1.01, 1.36]	0.82 [0.70, 0.95]	0.70 [0.60, 0.83]	0.69 [0.59, 0.81]
	M	1.01 [0.88, 1.17]	1.00 [0.86, 1.15]	0.96 [0.83, 1.11]	0.63 [0.54, 0.74]	0.56 [0.48, 0.66]	0.55 [0.47, 0.65]

BHPS=British Household Panel; ELSA=English Longitudinal Study of Ageing; NSHD=National Survey of Health and Development; FPS=Finnish Public Sector Study; GAZEL=Electricité De France-Gaz De France; HRS=Health and Retirement Study.

†Analyses involving NSHD, a birth cohort, were not adjusted for birth cohort.

Supplementary Table 5: Hazard ratios (HR) for low education (vs. high) and risk of 'health-related' exit from work

		Full sample (ages 40-75)			Restricted sample (ages 40-65)		
		Unadjusted	+ adjustment for birth cohort†	+ adjustment for poor self-rated health	Unadjusted	+ adjustment for birth cohort†	+ adjustment for poor self-rated health
		HR [95% C.I.]	HR [95% C.I.]	HR [95% C.I.]	HR [95% C.I.]	HR [95% C.I.]	HR [95% C.I.]
BHPS	F	2.11 [1.44, 3.09]	2.24 [1.52, 3.30]	1.94 [1.31, 2.86]	2.12 [1.45, 3.12]	2.24 [1.52, 3.30]	1.97 [1.33, 2.91]
	M	2.43 [1.60, 3.69]	2.49 [1.63, 3.80]	2.21 [1.44, 3.39]	2.16 [1.41, 3.29]	2.14 [1.39, 3.30]	1.86 [1.20, 2.88]
ELSA	F	1.66 [1.00, 2.76]	1.74 [1.04, 2.88]	1.62 [0.97, 2.70]	1.84 [1.09, 3.12]	1.86 [1.10, 3.16]	1.87 [1.11, 3.18]
	M	3.03 [1.56, 5.88]	3.02 [1.55, 5.87]	2.44 [1.25, 4.79]	3.20 [1.64, 6.24]	3.10 [1.58, 6.07]	2.62 [1.33, 5.16]
FPS	F	2.34 [2.15, 2.56]	2.36 [2.16, 2.57]	2.24 [2.06, 2.45]	2.34 [2.14, 2.55]	2.35 [2.15, 2.56]	2.24 [2.05, 2.44]
	M	3.55 [2.99, 4.20]	3.55 [2.99, 4.20]	3.09 [2.60, 3.67]	3.52 [2.97, 4.18]	3.52 [2.97, 4.17]	3.05 [2.57, 3.62]
GAZEL	F	1.30 [0.77, 2.21]	1.38 [0.81, 2.34]	1.32 [0.78, 2.24]	1.31 [0.77, 2.22]	1.38 [0.81, 2.35]	1.32 [0.78, 2.24]
	M	3.53 [2.32, 5.39]	3.88 [2.54, 5.92]	3.47 [2.27, 5.28]	3.53 [2.31, 5.38]	3.87 [2.54, 5.90]	3.46 [2.27, 5.27]
HRS	F	2.39 [1.53, 3.74]	2.83 [1.79, 4.46]	2.09 [1.31, 3.33]	2.34 [1.44, 3.80]	2.32 [1.41, 3.82]	1.94 [1.17, 3.21]
	M	2.51 [1.63, 3.86]	2.89 [1.87, 4.49]	2.44 [1.57, 3.81]	2.79 [1.77, 4.41]	2.67 [1.67, 4.26]	2.23 [1.39, 3.59]
NSHD	F	2.29 [1.00, 5.26]	2.29 [1.00, 5.26]	2.23 [0.97, 5.11]	1.81 [0.79, 4.15]	1.81 [0.79, 4.15]	1.76 [0.77, 4.04]
	M	2.70 [1.39, 5.27]	2.70 [1.39, 5.27]	2.53 [1.30, 4.94]	2.28 [1.09, 4.78]	2.28 [1.09, 4.78]	2.17 [1.03, 4.54]
Whitehall II	F	2.07 [1.37, 3.12]	1.83 [1.20, 2.77]	1.83 [1.21, 2.78]	1.89 [1.24, 2.87]	1.52 [0.99, 2.35]	1.54 [1.00, 2.36]
	M	1.64 [1.25, 2.14]	1.54 [1.18, 2.02]	1.52 [1.16, 1.99]	1.54 [1.17, 2.02]	1.32 [1.00, 1.74]	1.30 [0.99, 1.71]

BHPS=British Household Panel; ELSA=English Longitudinal Study of Ageing; NSHD=National Survey of Health and Development; FPS=Finnish Public Sector Study; GAZEL=Electricité De France-Gaz De France; HRS=Health and Retirement Study.

†Analyses involving NSHD, a birth cohort, were not adjusted for birth cohort.

Supplementary Table 6: Hazard ratios (HR) for low occupational grade (vs. high) and risk of 'health-related' exit from work

		Full sample (ages 40-75)			Restricted sample (ages 40-65)		
		Unadjusted	+ adjustment for birth cohort	+ adjustment for poor self-rated health	Unadjusted	+ adjustment for birth cohort	+ adjustment for poor self-rated health
		HR [95% C.I.]	HR [95% C.I.]	HR [95% C.I.]	HR [95% C.I.]	HR [95% C.I.]	HR [95% C.I.]
BHPS	F	2.55 [1.53, 4.23]	2.60 [1.56, 4.32]	2.23 [1.34, 3.73]	2.62 [1.58, 4.36]	2.66 [1.60, 4.43]	2.33 [1.40, 3.89]
	M	2.42 [1.53, 3.83]	2.40 [1.51, 3.79]	2.29 [1.45, 3.64]	2.36 [1.49, 3.72]	2.33 [1.47, 3.69]	2.15 [1.36, 3.40]
ELSA	F	2.16 [1.44, 3.25]	2.22 [1.48, 3.33]	2.04 [1.36, 3.07]	2.15 [1.42, 3.26]	2.17 [1.43, 3.28]	1.97 [1.30, 2.99]
	M	2.67 [1.67, 4.26]	2.64 [1.65, 4.22]	2.38 [1.48, 3.81]	2.66 [1.66, 4.26]	2.50 [1.56, 4.01]	2.21 [1.38, 3.55]
FPS	F	3.59 [3.25, 3.97]	3.60 [3.26, 3.98]	3.42 [3.09, 3.78]	3.57 [3.23, 3.94]	3.57 [3.23, 3.95]	3.39 [3.07, 3.75]
	M	3.63 [3.07, 4.30]	3.63 [3.07, 4.30]	3.12 [2.64, 3.70]	3.60 [3.04, 4.25]	3.59 [3.04, 4.25]	3.10 [2.62, 3.67]
GAZEL	F	3.85 [1.95, 7.59]	4.04 [2.05, 7.97]	3.39 [1.72, 6.70]	3.83 [1.94, 7.56]	4.01 [2.03, 7.91]	3.37 [1.71, 6.66]
	M	5.43 [3.77, 7.83]	5.34 [3.71, 7.69]	4.60 [3.19, 6.63]	5.44 [3.77, 7.83]	5.34 [3.71, 7.70]	4.60 [3.19, 6.63]
HRS	F	2.75 [1.74, 4.36]	2.88 [1.82, 4.56]	2.41 [1.51, 3.83]	3.07 [1.86, 5.06]	3.03 [1.83, 5.01]	2.80 [1.69, 4.64]
	M	2.77 [1.70, 4.51]	2.89 [1.77, 4.71]	2.39 [1.46, 3.91]	2.38 [1.42, 3.99]	2.30 [1.37, 3.86]	1.96 [1.17, 3.30]
NSHD	F	1.73 [1.08, 2.76]	1.73 [1.08, 2.76]	1.66 [1.04, 2.65]	1.35 [0.84, 2.20]	1.35 [0.84, 2.20]	1.31 [0.81, 2.13]
	M	1.62 [0.91, 2.91]	1.62 [0.91, 2.91]	1.61 [0.90, 2.89]	1.70 [0.94, 3.08]	1.70 [0.94, 3.08]	1.68 [0.93, 3.05]
Whitehall II	F	3.70 [2.10, 6.52]	3.40 [1.92, 6.02]	2.80 [1.57, 4.97]	2.80 [1.55, 5.07]	2.29 [1.26, 4.18]	1.81 [0.99, 3.31]
	M	5.33 [3.81, 7.45]	5.11 [3.66, 7.15]	3.86 [2.74, 5.44]	3.53 [2.49, 5.02]	3.11 [2.18, 4.44]	2.45 [1.71, 3.52]

BHPS=British Household Panel; ELSA=English Longitudinal Study of Ageing; NSHD=National Survey of Health and Development; FPS=Finnish Public Sector Study; GAZEL=Electricité De France-Gaz De France; HRS=Health and Retirement Study.

[†]Analyses involving NSHD, a birth cohort, were not adjusted for birth cohort.

Supplementary Table 7: Hazard ratios from Cox proportional hazards models comparing alternative (a) measures of health exit and (b) study designs

	Men				Wor	
	Any exit from work		Health-related exit from work		Any exit from work	
	Low education (vs. high)	Low occupational grade (vs. high)	Low education (vs. high)	Low occupational grade (vs. high)	Low education (vs. high)	Low occupational grade (vs. high)
	Adjusted† HR [95% C.I.]	Adjusted† HR [95% C.I.]	Adjusted† HR [95% C.I.]	Adjusted† HR [95% C.I.]	Adjusted† HR [95% C.I.]	Adjusted† HR [95% C.I.]
Study design‡						
General population survey	1.25 [1.14, 1.36]	1.21 [1.12, 1.30]	2.74 [2.09, 3.59]	2.57 [1.96, 3.38]	1.36 [1.26, 1.47]	1.18 [1.10, 1.27]
Occupational cohort	1.61 [1.55, 1.68]	1.16 [1.11, 1.20]	2.17 [1.90, 2.49]	4.71 [4.12, 5.39]	1.26 [1.21, 1.31]	1.19 [1.14, 1.24]
Birth cohort	0.99 [0.80, 1.21]	0.89 [0.70, 1.13]	2.53 [1.30, 4.94]	1.61 [0.90, 2.89]	1.18 [0.89, 1.55]	1.15 [0.94, 1.40]
Wald test χ^2 (df, p-value)	402.9 (4, p<0.000)	165.2 (4, p<0.000)	3.9 (4, p=0.42)	30.5 (4, p<0.000)	44.4 (4, p<0.000)	52.0 (4, p<0.000)
Assessment of health exit						
Register-based	Not applicable.		2.91 [2.48, 3.40]	4.58 [3.93, 5.33]	Not applicable.	
Self-report			2.07 [1.73, 2.47]	2.61 [2.17, 3.14]		
Wald test χ^2 (df, p-value)			4.2 (2, p=0.13)	36.0 (2, p<0.000)		

†Adjusted for poor self-rated health and birth cohort, except for analyses involving NSHD, which were adjusted for poor self-rated health only.

‡ General population survey = BHPS, ELSA, HRS; occupational cohort = FPS, GAZEL, Whitehall II; birth cohort = NSHD.

BHPS=British Household Panel; ELSA=English Longitudinal Study of Ageing; NSHD=National Survey of Health and Development; FPS=Finnish Public Sector Study; C
France; HRS=Health and Retirement Study.

nen

Health-related exit from work	
Low education (vs. high)	Low occupational grade (vs. high)
Adjusted† HR [95% C.I.]	Adjusted† HR [95% C.I.]
2.75 [2.14, 3.52]	2.86 [2.20, 3.70]
1.79 [1.65, 1.94]	3.06 [2.78, 3.38]
2.23 [0.97, 5.11]	1.66 [1.04, 2.65]
32.1 (4, p<0.000)	15.4 (4, p=0.003)
1.97 [1.81, 2.15]	3.27 [2.96, 3.61]
2.60 [2.14, 3.16]	2.36 [1.94, 2.86]
20.6 (2, p<0.000)	14.1 (2, p<0.000)

GAZEL=Electricité De France-Gaz De

Supplementary material: Question wording for self-rated health

	Wording of question and responses	Definition of poor health
BHPS	Please think back over the last 12 months about how your health has been. Compared to people of your own age, would you say that your health has on the whole been… “Excellent”, “Good”, “Fair”, “Poor”, “Very poor”.	“Poor” or “Very poor”
ELSA	Would you say your health is… “Excellent”, “Very good”, “Good”, “Fair”, “Poor”.	“Poor”
NSHD	“How is your health in general?” “Excellent”, “Very good”, “Good”, “Fair”, “Poor”.	“Poor”
Whitehall II	“In general, would you say your health is:” “Excellent”, “Very good”, “Good”, “Fair”, “Poor”.	“Fair” or “Poor”
FPS	“How do you rate your health?” “Excellent”, “Good”, “Average”, “Rather poor”, “Poor”.	“Fairly poor”, “Poor”
GAZEL	“How would you describe your general health” A (“Very good”) through H (“Very poor”).	F, G, or H (“Very poor”)
HRS	“Would you say your health is...” “Excellent”, “Very good”, “Good”, “Fair”, “Poor”.	“Poor”
